# Supplementary material for: Oxidation-State Dynamics and Emerging Patterns in Magnetite
Source: J Phys Chem Lett. 2023 Jul 21;14(30):6800–7. doi: 10.1021/acs.jpclett.3c01290 (PMC10405268; doi:10.1021/acs.jpclett.3c01290)
Supplement: Supplementary file 2 — jz3c01290_si_002.pdf [file jz3c01290_si_002.pdf]

Supporting Information: Oxidation State Dynamics and Emerging Patterns in Magnetite

Emre Gürsoy
Institute of Polymers and Composites, Hamburg University of Technology, 21073 Hamburg, Germany

Gregor B. Vonbun-Feldbauer
Institute of Advanced Ceramics, University of Technology, 21073 Hamburg, Germany

Robert H. Meißner*
Institute of Polymers and Composites, Hamburg University of Technology, 21073 Hamburg, Germany and Institute of Surface Science, Helmholtz-Zentrum Hereon, 21502 Geesthacht, Germany

METHODS

Density Functional Theory Calculations

The Density Functional Theory (DFT) calculations employed here follow the setup of previous studies[1–3] in which the Vienna Ab Initio Simulation Package (VASP, version 5.4.4)[4,5] with the PBE+U approach[6,7] ($U_{\text{eff}} = 4\,\text{eV}$ on Fe d-electrons) and PAW pseudopotentials[8] was used for studying magnetite surfaces. The oxidation states of the ions are approximated via Bader charge analyses which were performed using the implementation of Henkelman *et al.*[9–11] Formal oxidation states of Fe^{2+} and Fe^{3+} have been assigned in the literature to the Fe ions based on charge disproportionations of about $0.3e$.[12] The clustering of iron atoms in the present work according to their *ab initio* derived Bader charges into Fe^{2+} and Fe^{3+} leads to a clear distinction between these two species with a difference between the mean Bader charges of both groups of about $0.3e$. This value is found for all systems considered. Due to the different chemical environments for octahedral and tetrahedral coordinated iron atoms and in the vicinity of surfaces, there is a spread in the charges within each species. However, these spreads are significantly smaller than the difference between the two species. Interestingly, the spreads are larger for (111) surfaces of about $0.15e$ than for (001) surfaces of about $0.07e$. The spreads are typically larger for Fe^{3+} than for Fe^{2+} because the former species usually contains octahedral and tetrahedral irons, while the latter species contains only octahedral irons. However, the minimum distance of the charges of both species is always at least $0.2e$, which allows a clear distinction.

In the previous publications mentioned above, the charge distributions and inferred oxidation states, however, were rather strongly constrained by the system size and symmetry used in the calculations. Here, larger systems with surface unit cells up to 3×3 and up to 37 atomic layers for the slab thickness were used. However, because of computational limitations and convergence issues for larger systems not all of the combinations of surface terminations and different surface cell as well as slab thickness were treated. Particularly, the thickness and the surface cell size were not maximized at the same time and thick slabs tend to be difficult to converge. Symmetries other than time-reversal symmetry were disabled in the calculations. Particularly, turning off symmetry causes subtle changes in the charge distribution which are, however, important for obtaining the oxidation states. While disabling symmetry for all system sizes tested resulted in a realistic charge distribution with two distinct iron oxidation states, the charge distribution when symmetry was used often depended on the system dimensions and in some cases resulted in all atoms in a layer having the same charge, while not allowing charge neutrality with two oxidation states but requiring a third $Fe^{2.5+}$-like state.

Molecular Dynamics Simulations

All force field simulations are performed using LAMMPS.[13] Molecular dynamics (MD) simulations are performed in the NVT ensemble. The canonical ensemble (NVT) defines a statistical ensemble that represents all accessible states of a closed system at a constant number of particles N, volume V and temperature T. Cubic nanoparticles (NPs) have been produced by simple slicing from a bulk magnetite structure Fd$\bar{3}$m, resulting in 1466 atoms and an edge length of 2.3 nm. Spherical magnetite NPs were then generated using the same crystal structure via NanoCrystal.[14] If required, a Nosé-Hoover thermostat (NVT) is used to maintain the system at the desired temperature, with relaxation time constants of 100 time units for the thermostat. The equations of motion are integrated with a time

TABLE S1. Force field parameters of non-bonded interactions describing magnetite.[18] Partial charge of Fe^{2+} has not been mentioned elsewhere for magnetite, but is chosen here to correspond to the opposite charge of oxygen.

Atom type	ϵ_i / kcal mol^{-1}	σ_i / Å	q / e
Fe^{2+}	$9.0298 \cdot 10^{-7}$	4.90620	1.050
Fe^{3+}	$9.0298 \cdot 10^{-7}$	4.90620	1.575
O	0.1554	3.16554	-1.050

step of 0.5 fs using the velocity Verlet algorithm. This relatively small timestep is required for the hybrid Monte Carlo / Molecular Dynamics (MC/MD) approach. Long-range Coulomb interactions are treated either explicitly in the case of nanoparticles or with a PPPM[15] solver with a (relative) accuracy of 10^{-6} and a real-space cutoff of 12 Å for bulk and slab systems and a real-space cutoff of 35 Å for nanoparticles. For bulk: p p p boundaries, for surfaces: p p f boundaries and for nanoparticles: f f f boundaries are used (p stands for periodic and f for non-periodic and fixed). To eliminate problems due to periodicity in slab calculations, a sufficient amount of vacuum, i.e. three times the box size in the non-periodic dimension, has been added together with the appropriate dipole correction.[16] Van der Waals interactions are truncated at 12 Å.

Magnetite Force Field

Parameters to model magnetite are taken from the ClayFF force field.[17,18] ClayFF offers a robust parameterization for many minerals and their interfaces to aqueous solutions. For solids with ionic character such as magnetite, it is often sufficient to represent the potential energy only by simple, non-bonded interactions, i.e., electrostatic and van der Waals interactions. Short-ranged van der Waals interactions are usually modeled by a Lennard-Jones potential, while Coulomb's law between point charges is used for electrostatic interactions:

$$U_{\mathrm{non-bonded}} = \sum_{ij} 4\epsilon_{ij} \left[\left(\frac{\sigma_{ij}}{r_{ij}} \right)^{12} - \left(\frac{\sigma_{ij}}{r_{ij}} \right)^{6} \right] + \frac{q_i q_j}{r_{ij}} \tag{S1}$$

q_i and q_j denote the atomic partial point charges between interacting pairs of atoms i and j. Distances between interacting atoms are denoted by r_{ij}. ϵ_{ij} indicates how strongly these atoms attract each other and $\sqrt[6]{2}\sigma_{ij}$ corresponds to the distance at which the potential between the atoms has its minimum. For convenience, the parameters used here to describe magnetite are summarized in Table S1. Pair coefficients ϵ_{ij} and σ_{ij} for interacting atom pairs are calculated by standard Lorentz-Berthelot mixing rules: $\epsilon_{ij} = \sqrt{\epsilon_i \epsilon_j}$, $\sigma_{ij} = \frac{1}{2}(\sigma_i + \sigma_j)$.

Oxidation State Swaps

The accuracy of a force field depends largely on whether atomic charges have been parameterized for the particular electrostatic environment, i.e., our case, for the oxidation states in magnetite. Previously, this required a new set of charges for each surface termination of (001)-DBT, (001)-SCV, or (111)-tet1 and could also depended on whether adsorbed water or ligands are present, each yielding a different electrostatic configuration.[1] To avoid having to determine atomic charges for each magnetite configuration, we propose an Oxidation Swap Method (OSM). Although it is a rather simple approach, it works surprisingly well and even allows us to investigate many of the recent findings about magnetite, i.e. polarons, to some extent.

Until not otherwise noted, oxidation swaps are generally allowed between all iron sites, i.e., between octahedral and tetrahedral sites. Swapping only between octahedral iron sites is computationally more efficient, but when surfaces are involved and there are undercoordinated iron atoms, as observed for example in the case of the (111) surface where the oxidation state of a surface Fe_{tet} seems to resemble rather an oxidation state of Fe^{2+},[1] a more general approach is promising to capture this as well.

Unfortunately, finding the global minimum by simulated annealing is not straightforward and requires often fine-tuning of the annealing procedure.[19] The annealing scheme proposed byKirkpatrick et al.[20] consists of three components: (i) an initial temperature, T_0^{MC}, which should be high enough to allow all possible oxidation state changes between Fe ions; (ii) an exponential temperature decrease function, here $T_\lambda^{\mathrm{MC}} = T_0^{\mathrm{MC}} \cdot \eta^\lambda$, where η determines the exponential decrease with each MC cycle denoted by λ; (iii) the number of swaps, n_{swaps}, which should be chosen

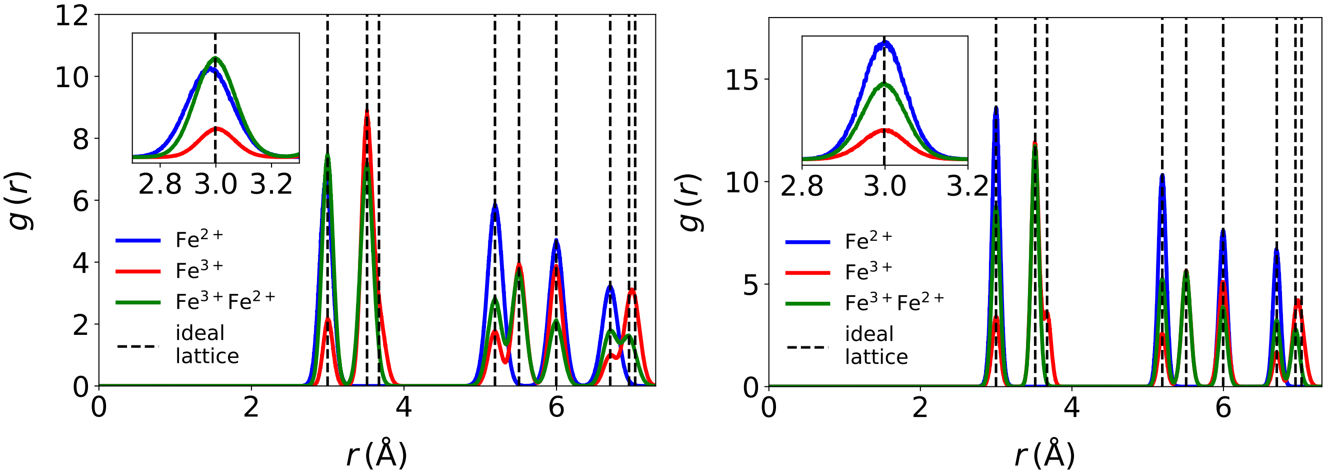

FIG. S1. Radial distribution functions (RDFs) from hybrid MC/MD simulations: (left) T^{MC} held below the critical MC temperature $T_{\mathrm{c}}^{\mathrm{MC}}$; (right) T^{MC} held above the critical MC temperature. In addition, the RDF of the ideal (i.e. undistorted) lattice is shown by dashed black lines. Insets show enlarged RDF of the first peak. Corresponding iron species are indicated in legend.

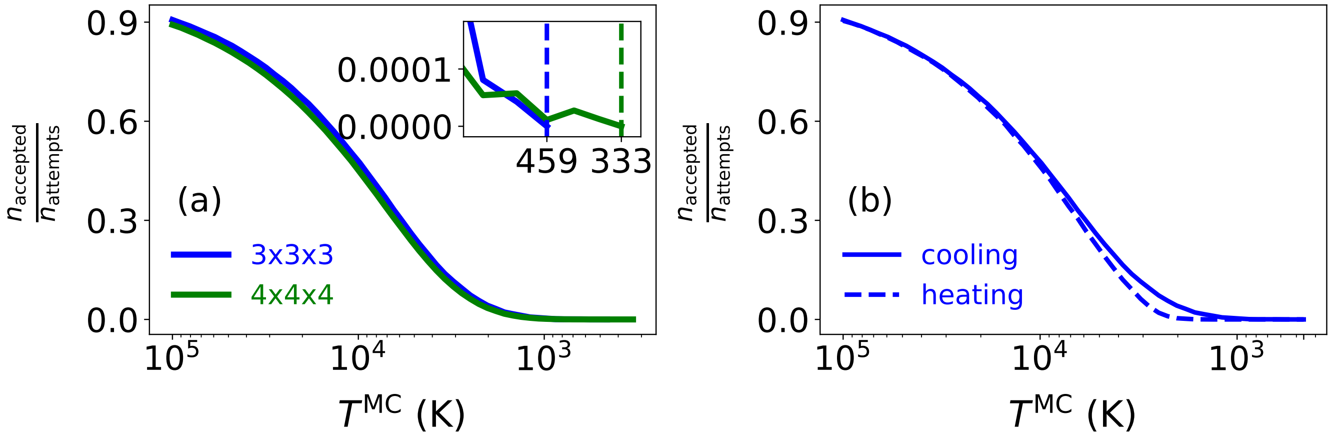

FIG. S2. (a) Swap probabilities of two different sized bulk magnetite structures ($3 \times 3 \times 3$ and $4 \times 4 \times 4$) with respect to T^{MC}. Critical MC temperatures where swaps basically stop are highlighted in the inset, i.e. 459 K for $3 \times 3 \times 3$ and 333 K for $4 \times 4 \times 4$. (b) Swap probability obtained by first cooling (solid line), then applying energy minimisation and reheating (dashed line) a $3 \times 3 \times 3$ bulk magnetite.

large enough to theoretically allow exchanges between all relevant iron sites each cycle – ideally multiple times. Consequently, if oxidation states are randomly distributed among the irons in a bulk magnetite system, minimization with our oxidation swapping should yield $n_{\mathrm{Fe}_{\mathrm{oct}}^{2+}}/n_{\mathrm{Fe}_{\mathrm{oct}}^{3+}} = 1$. The initial temperature of the simulated annealing is $T_0^{\mathrm{MC}} = 10^5\,\mathrm{K}$, which corresponds roughly to a swapping probability of 0.9 for all systems. We chose a relatively high temperature decrease coefficient using the relationship $\eta = \left(T_{\mathrm{f}}^{\mathrm{MC}}/T_0^{\mathrm{MC}}\right)^{1/n_{\mathrm{Fe}}}$ to ensure, that the temperature decrements between the initial temperature T_0^{MC} and the final temperature $T_{\mathrm{f}}^{\mathrm{MC}}$ (usually 1 K) are not too large to prevent the system from getting stuck in a local minima.

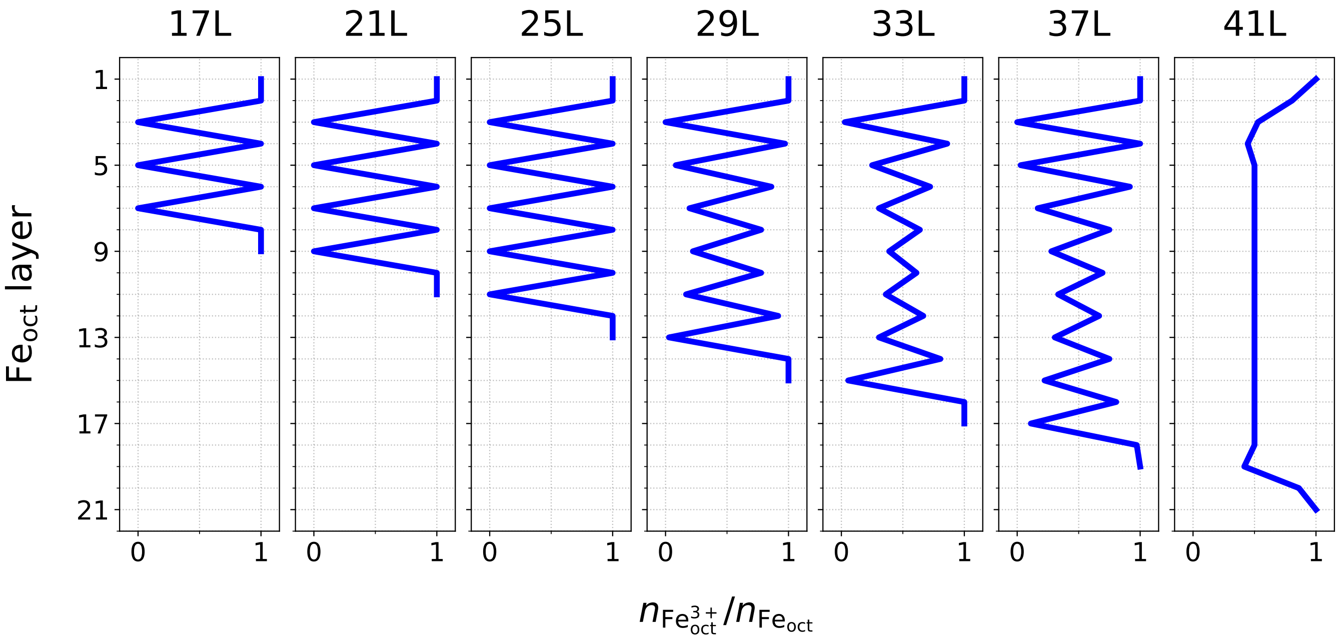

FIG. S3. Ratio of Fe^{3+}_{oct} ions in each octahedral layer, $n_{Fe^{3+}_{oct}}/n_{Fe_{oct}}$ for minimised oxidation state configuration of (001)-DBT surfaces with respect to the corresponding octahedral layer. The number of atomic layers is given at the top of each figure.

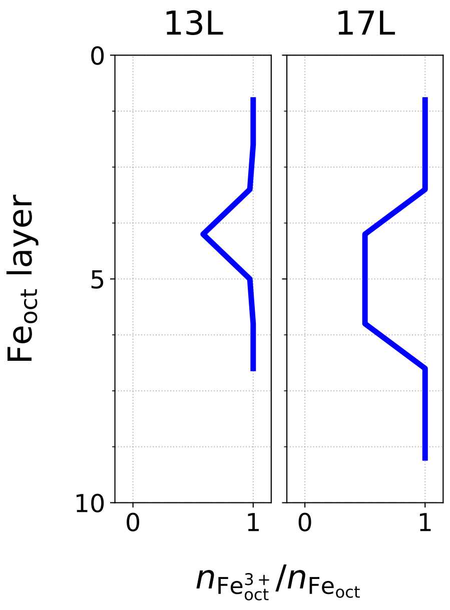

FIG. S4. Ratio of Fe^{3+}_{oct} ions in each octahedral layer, $n_{Fe^{3+}_{oct}}/n_{Fe_{oct}}$ for minimised oxidation state configuration of (001)-SCV surfaces with respect to the corresponding octahedral layer. The number of atomic layers is given at the top of each figure.

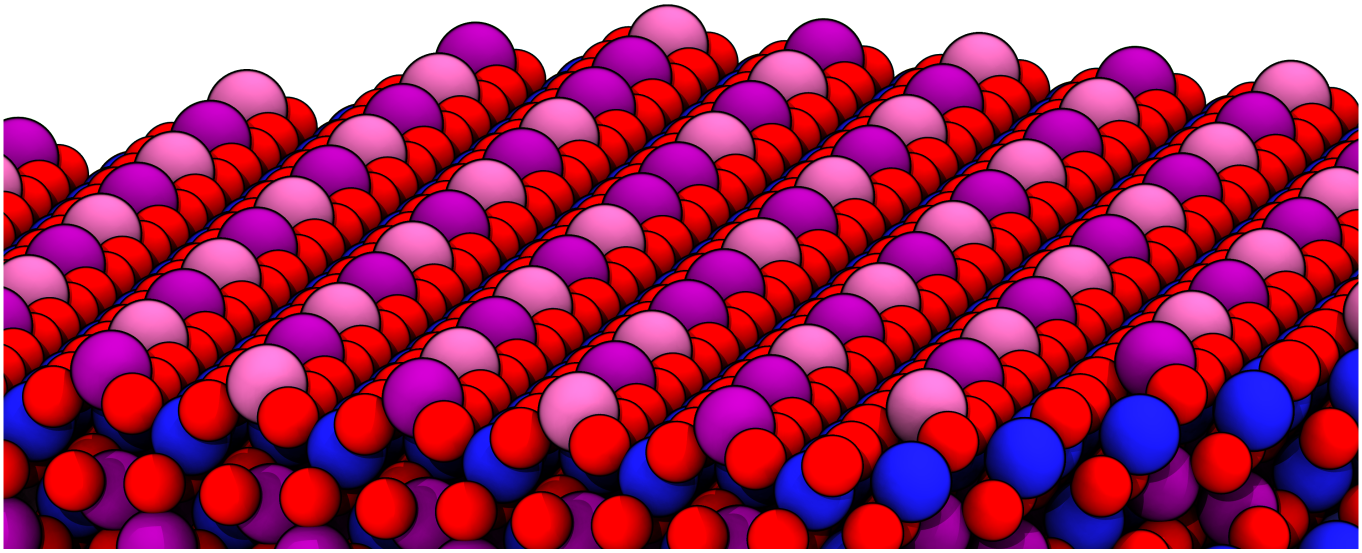

FIG. S5. Minimized oxidation state configuration of 41 layer (111)-tet1 magnetite surface. Color code: Fe^{3+}_{tet} – purple, Fe^{2+}_{tet} – mauve, Fe^{3+}_{oct} – blue, Fe^{2+}_{oct} – light blue.

* robert.meissner@tuhh.de

(1) Konuk, M.; Sellschopp, K.; Vonbun-Feldbauer, G. B.; Meißner, R. H. Modeling Charge Redistribution at Magnetite Interfaces in Empirical Force Fields. *J. Phys. Chem. C* **2021**, *125*, 4794–4805.

(2) Arndt, B.; Sellschopp, K.; Creutzburg, M.; Grånäs, E.; Krausert, K.; Vonk, V.; Müller, S.; Noei, H.; Feldbauer, G. B. V.; Stierle, A. Carboxylic acid induced near-surface restructuring of a magnetite surface. *Commun. Chem.* **2019**, *2*.

(3) Creutzburg, M.; Sellschopp, K.; Tober, S.; Grånäs, E.; Vonk, V.; Mayr-Schmölzer, W.; Müller, S.; Noei, H.; Vonbun-Feldbauer, G. B.; Stierle, A. Heterogeneous adsorption and local ordering of formate on a magnetite surface. *J. Phys. Chem. Lett.* **2021**, *12*, 3847–3852.

(4) Kresse, G.; Furthmüller, J. Efficient Iterative Schemes for Ab Initio Total-Energy Calculations Using a Plane-Wave Basis Set. *Phys. Rev. B* **1996**, *54*, 11169–11186.

(5) Kresse, G.; Furthmüller, J. Efficiency of Ab-Initio Total Energy Calculations for Metals and Semiconductors Using a Plane-Wave Basis Set. *Comput. Mater. Sci.* **1996**, *6*, 15–50.

(6) Perdew, J. P.; Burke, K.; Ernzerhof, M. Generalized Gradient Approximation Made Simple. *Phys. Rev. Lett.* **1996**, *77*, 3865–3868.

(7) Dudarev, S. L.; Botton, G. A.; Savrasov, S. Y.; Humphreys, C. J.; Sutton, A. P. Electron-Energy-Loss Spectra and the Structural Stability of Nickel Oxide: An LSDA+U Study. *Phys. Rev. B* **1998**, *57*, 1505–1509.

(8) Blöchl, P. E. Projector Augmented-Wave Method. *Phys. Rev. B* **1994**, *50*, 17953–17979.

(9) Henkelman, G.; Arnaldsson, A.; Jónsson, H. A fast and robust algorithm for Bader decomposition of charge density. *Comput. Mater. Sci.* **2006**, *36*, 354–360.

(10) Sanville, E.; Kenny, S. D.; Smith, R.; Henkelman, G. Improved grid-based algorithm for Bader charge allocation. *J. Comput. Chem.* **2007**, *28*, 899–908.

(11) Tang, W.; Sanville, E.; Henkelman, G. A grid-based Bader analysis algorithm without lattice bias. *J. Phys. Condens. Matter* **2009**, *21*, 084204.

(12) Liu, H.; Di Valentin, C. Band Gap in Magnetite above Verwey Temperature Induced by Symmetry Breaking. *J. Phys. Chem. C* **2017**, *121*, 25736–25742.

(13) Plimpton, S. Fast Parallel Algorithms for Short-Range Molecular Dynamics. *J. Comput. Phys.* **1995**, *117*, 1–19.

(14) Chatzigoulas, A.; Karathanou, K.; Dellis, D.; Cournia, Z. NanoCrystal: A Web-Based Crystallographic Tool for the Construction of Nanoparticles Based on Their Crystal Habit. *J. Chem. Inf. Model.* **2018**, *58*, 2380–2386.

(15) Hockney, R.; Eastwood, J. *Computer Simulation Using Particles*; CRC Press, 2021.

(16) Yeh, I.-C.; Berkowitz, M. L. Ewald summation for systems with slab geometry. *J. Chem. Phys.* **1999**, *111*, 3155–3162.

(17) Cygan, R. T.; Liang, J.-J.; Kalinichev, A. G. Molecular models of hydroxide, oxyhydroxide, and clay phases and the development of a general force field. *J. Phys. Chem. B* **2004**, *108*, 1255–1266.

(18) Cygan, R. T.; Greathouse, J. A.; Kalinichev, A. G. Advances in Clayff Molecular Simulation of Layered and Nanoporous Materials and Their Aqueous Interfaces. *J. Phys. Chem. C* **2021**, *125*, 17573–17589.

(19) Ingber, L. Adaptive simulated annealing (ASA): Lessons learned. *arXiv preprint cs/0001018* **2000**,

(20) Kirkpatrick, S.; Gelatt, C. D.; Vecchi, M. P. Optimization by Simulated Annealing. *Science* **1983**, *220*, 671–680.
